# Improvement of Visible Photodetection of Chemical Vapor Deposition-Grown MoS_2_ Devices via Graphene/Au Contacts

**DOI:** 10.3390/s22249687

**Published:** 2022-12-10

**Authors:** Yeongsik Hwa, Sang-Soo Chee

**Affiliations:** 1 Nano Convergence Materials Center, Korea Institute of Ceramic Engineering and Technology (KICET), Jinju 52851, Republic of Korea; 2 School of Materials Science and Engineering, Gyeongsang National University, Jinju 52828, Republic of Korea

**Keywords:** MoS_2_, graphene, interlayer, contact engineering, large area

## Abstract

Two-dimensional (2D) molybdenum disulfide (MoS_2_) is a promising material for constructing high-performance visible photosensor arrays because of its high mobility and scale-up process. These distinct properties enable the construction of practical optoelectrical sensor arrays. However, contact engineering for MoS_2_ films is not still optimized. In this work, we inserted a graphene interlayer between the MoS_2_ films and Au contacts (graphene/Au) via the wet-transfer method to boost the device performance. Using graphene/Au contacts, outstanding electrical properties, namely field-effect mobility of 12.06 cm^2^/V∙s, on/off current ratio of 1.0 × 10^7^, and responsivity of 610 A/W under illumination at 640 nm, were achieved. These favorable results were from the Fermi-level depinning effect induced by the graphene interlayer. Our results may help to construct large-area photonic sensor arrays based on 2D materials.

## 1. Introduction

Two-dimensional (2D) molybdenum disulfide (MoS_2_) is a promising next-generation material owing to its superior electrical and photoelectrical properties in comparison to those of its counterparts [1,2]. Moreover, its optimized scale-up production and layer-dependent bandgap (approximately 1.4–2 eV) enable the construction of practical optoelectrical visible photosensor arrays [1,2,3].

Optimal contact engineering is needed to construct practical and large-area photonic sensor arrays based on MoS_2_ because the Schottky barrier height (SBH) formed by the semiconducting MoS_2_ and metal contacts is a critical factor that determines the electrical and photoelectrical properties of the resultant devices [4]. Many studies on the metal-contact engineering of MoS_2_ have been conducted [4,5,6]. Das et al. reported that scandium, an extremely low work-function metal, provides improved electrical performance with good mobility (~700 cm^2^/V∙s) due to lowered SBHs [4]. However, they observed a high difference between the experimental SBH and the theoretical one calculated using the Schottky–Mott rule. This discrepancy was attributed to the metal-induced Fermi-level pinning effect, which suffers from the construction of high-performance 2D devices [7,8]. To eradicate this effect, many studies on contact engineering have been conducted, such as interlayer and phase transition from a semiconducting to metallic properties [9,10]. The graphene interlayer is the most practical solution among the proposed constructs because its crystal structure is similar to MoS_2_ and therefore can prevent the interaction between MoS_2_ and metal contacts [9,11,12]. Owing to this distinctive characteristic, several studies on graphene interlayer contacts in MoS_2_ devices have been reported, realizing the Fermi-level depinning that improves the electrical and photoelectrical properties [9,11,13]. However, these studies mainly utilized small MoS_2_ and graphene flakes obtained from mechanical exfoliation [9,11,14,15,16], and this strategy cannot be applied to large-area device arrays because of the limited flake size. Thus, it is necessary to find an alternative approach to construct graphene interlayers for large-area MoS_2_ device arrays with practical photoelectronic applications and good performance.

Here, we inserted a graphene interlayer between large-area MoS_2_ films grown by chemical vapor deposition (CVD) and Au contacts (graphene/Au contacts) to induce Fermi-level depinning leading to the enhancement in device performance of the resultant MoS_2_ device arrays. To investigate the effect of the graphene interlayer, we also compare the performance of conventional Au contacts in terms of electrical and photoelectrical properties.

## 2. Materials and Methods

### 2.1. Synthesis of MoS_2_ and Graphene Monolayer Films

We first synthesized MoS_2_ film via CVD using precursor powders, including MoO_3_ (99.999%, Advanced Chemicals, Altona, Australia) and sulfur (99.9% Sigma Aldrich, St. Louis, MO, USA). Each precursor was placed into two ceramic crucibles. A MoO_3_ crucible was placed at the center of a 1 inch quartz tube, and a sulfur crucible was positioned upstream of the MoO_3_ crucible. A Si/SiO_2_ substrate was loaded downstream of the MoO_3_ crucible. Then, the furnace was heated to 650 °C and maintained at this temperature for 40 min with a flow of Ar (50 sccm) under 0.3 Torr. After finishing the reaction, natural cooling was performed with 200 sccm of Ar flow. Graphene monolayer film was also synthesized on Cu foil (Alfa Aesar, Haverhill, MA, USA) via identical CVD. The specific synthetic process is explained in our previous report [13].

### 2.2. Fabrication of MoS_2_ Devices with Au and Graphene/Au Contacts

We used a standard photolithography method to fabricate MoS_2_ devices. MoS_2_ films were first prepared on Si/SiO_2_ substrate. To construct patterned graphene, first, photolithography and oxygen plasma etching treatment were performed. Once the patterned graphene was prepared, it was transferred onto MoS_2_ film via the wet-transfer method, where we used a buffered oxide etch solution to delaminate graphene. The second photolithography was carried out on patterned graphene electrodes, followed by metal deposition (50 nm-thick Au). To minimize the misalignment, the size of the Au electrodes was slightly bigger (200 × 200 μm) than that of graphene interlayers (150 × 150 μm). To prevent current leakage, channel definition was performed with a length of 14 μm and width of 40 μm using the third photolithography. Finally, the uncovered MoS_2_ area was etched using SF_6_ plasma treatment. We also fabricated MoS_2_ devices with Au-only contacts via the same procedure without a graphene layer to investigate the effect of the graphene interlayer.

### 2.3. Characterization

Atomic force microscopy (AFM, XE-100, Park Systems, Suwon, Republic of Korea) and Raman/photoluminescence (PL) spectroscopy (LabRAM HR Evolution, Horiba Jobin-Yvon, Bensheim, Germany) equipped with a 532 nm laser were utilized at room temperature to explore the physical properties of MoS_2_ and graphene film grown by CVD. Transmission electron microscopy (TEM) measurement was performed to investigate the crystal structure of the obtained MoS_2_ film. Electrical and photoelectrical properties were characterized using a semiconducting parameter analyzer (E5270B, Agilent Technologies, Santa Clara, CA, USA) with a 10^−6^ Torr vacuum at 80–300 K and light emitting diode (LED) visible light at 640 nm.

## 3. Results and Discussion

**Figure 1:**
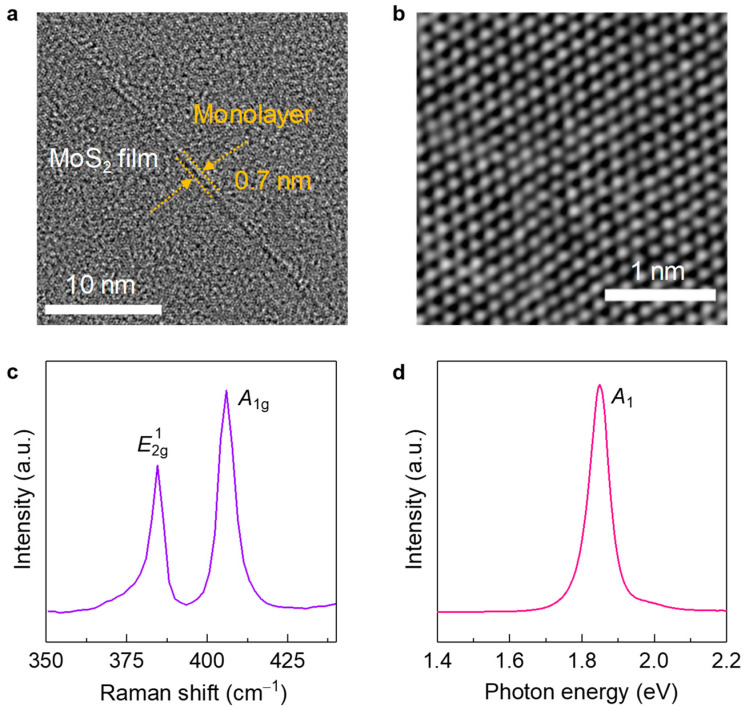
(**a**) TEM and (**b**) high-resolution STEM images of obtained MoS_2_ films grown by CVD. Scale bars denote 10 and 1 nm, respectively. (**c**) Raman and (**d**) PL spectra of our MoS_2_ films.

Figure 1a shows TEM images of MoS_2_ film synthesized via CVD. Overall, the obtained film shows good uniformity with no remarkable voids or cracks. (Figure 1a and Figure S1). In addition, the folded area indicates that the number of layers is monolayer. High-resolution scanning transmission electron microscopy (STEM) confirms a three-fold coordinated atomic arrangement [17], indicating that our MoS_2_ films correspond to a monolayer structure and are well grown via CVD (Figure 1b). Figure 1c,d show Raman and PL spectra of the obtained MoS_2_ films. In the Raman spectrum, there are two prominent peaks indicating $E_{2g}^{1}$ and *A*_1g_, involved in in- and out-of-plane modes [18,19], respectively (Figure 1c). From the Raman spectrum, we then calculated the peak difference (*A*_1g_ − $E_{2g}^{1}$) and full-width at half-maximum (FWHM) of $E_{2g}^{1}$, which indicate the number of layers and film uniformity, respectively [18,19]. The estimated peak difference and FWHM of $E_{2g}^{1}$ are 20.8 and 4.6 cm^−1^, respectively. These results further confirm that our MoS_2_ is a monolayer and that the film quality is comparable to that of a mechanically exfoliated flake [18,19]. In PL spectrum (Figure 1d), a distinctive peak at 1.81 eV corresponding to the A_1_ exciton can be seen. This observation further supports that our MoS_2_ film is a monolayer [19], which is consistent with the TEM images and Raman spectrum (Figure 1a–c).

**Figure 2:**
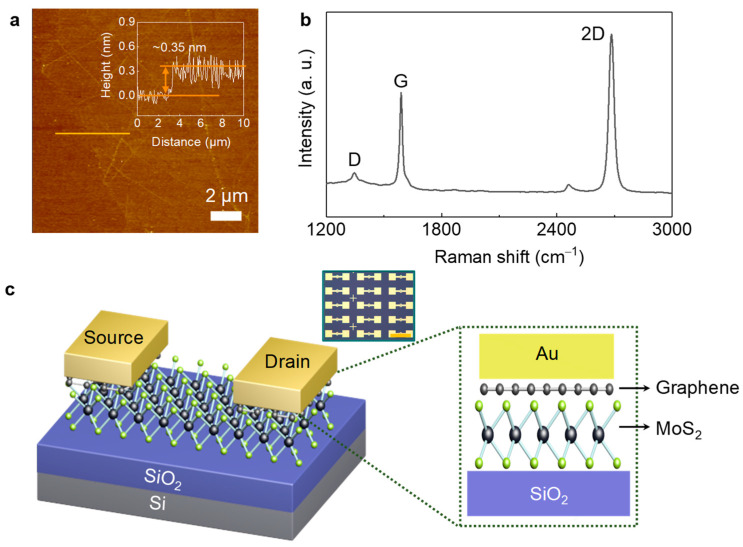
(**a**) AFM image and (**b**) Raman spectrum of graphene films synthesized by CVD. The wet-transfer process was used to transfer the films onto a Si/SiO_2_ substrate. (**c**) Schematic image of MoS_2_ devices with graphene/Au contacts (inset: optical microscopy image of MoS_2_ device arrays with graphene/Au contacts. Scale bar denotes 500 µm).

Figure 2a shows AFM image of wet-transferred graphene film on a Si/SiO_2_ substrate grown by CVD. Clear grain boundaries can be observed without noticeable defects or voids. The thickness of the obtained graphene film was estimated at approximately 0.35 nm (inset in Figure 2a), indicating the monolayer nature of the film. Raman spectroscopy measurement was performed to investigate the physical properties of synthesized graphene films (Figure 2b). Three peaks, namely D, G, and 2D peaks [13], can be observed. In particular, a strong 2D peak was observed, implying that our graphene film is a monolayer. Using this graphene monolayer film, we then fabricated MoS_2_ device arrays with graphene/Au contacts, as shown Figure 2c. The details are provided in the Materials and Methods section. Briefly, once the patterned graphene films were transferred onto MoS_2_ films via the wet-transfer method, source and drain electrodes were patterned using standard photolithography. Afterwards, metal deposition and channel definition were conducted. Our device fabrication enables realization of large-area MoS_2_ device arrays by using graphene interlayer contacts via CVD-grown films (inset in Figure 2c and Figure S1), contrary to the previous reports on only a single MoS_2_ device.

**Figure 3:**
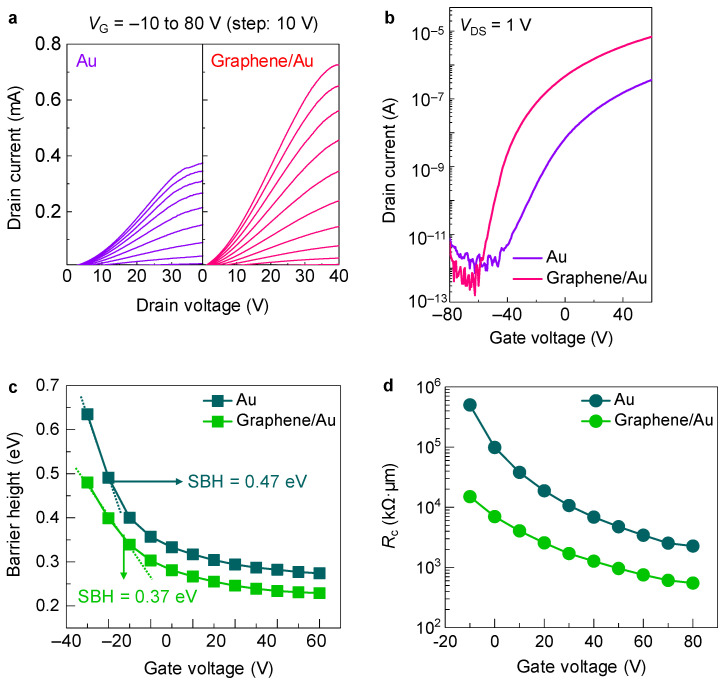
(**a**) Output and (**b**) transfer characterizations of MoS_2_ devices with Au and graphene/Au contacts. (**c**) Barrier height and (**d**) contact resistance (*R*_c_) of MoS_2_ devices with Au and graphene/Au contacts as a function of gate voltages.

Using the fabricated MoS_2_ devices with and without graphene/Au contacts, we measured electrical properties at room temperature under vacuum conditions. To achieve reliable data, we fabricated multiple MoS_2_ device arrays with and without a graphene layer. Both MoS_2_ devices showed identical *n*-type characteristics (Figure 3a,b). Note that on-state current levels of graphene-inserted devices were significantly enhanced compared to those of only Au-contacted devices. Such improvement in on-state current levels enhances the device performance, providing the average field-effect mobility of 12.06 cm^2^/V∙s (±1.34) and on/off current ratio of 1.01 × 10^7^(±2.52 × 10^6^), compared with those of Au-contacted devices (mobility: 2.1 cm^2^/V∙s (±0.75) and on/off current ratio: 1.0 × 10^6^ (±1.34 × 10^5^)), as shown Figure S2. In general, the improved mobility is accompanied with suppression of on/off current ratios while increasing off-state currents [20]. It is noteworthy that the on/off current ratio is improved in the case of our MoS_2_ devices with graphene/Au contacts while preserving off-state current levels. This enhancement presumably results from the decreased SBHs of the resultant devices and the Fermi-level depinning effect induced by the graphene interlayer, providing an efficient charge transfer between the graphene/Au contacts and MoS_2_ channels [13,21].

We calculated the SBHs by using a modified Richardson plot, as follows [22]:

$$\ln\left(\frac{I_{0}}{T^{2}}\right)-\left(\frac{q^{2}\sigma^{2}}{2k^{2}T^{2}}\right)=\ln\left(AA^{*}\right)-\frac{q\varphi_{B}}{kT}$$

where *T* is the temperature (K), *I*_0_ is the saturated current level, *q* is the electronic charge, *k* is the Boltzmann constant, *σ* is the standard deviation of the Gaussian function of the SBH, *A* is the device area, A* is the Richardson constant, and *φ_B_* is the barrier height. The Richardson constant is strongly associated with the effective mass of the material. It is estimated as A* = 4πqm*k^2^/*h*^3^, where m* is the effective mass of MoS_2_ and *h* is Planck’s constant [22,23]. From these equations, SBHs can be extracted by linear fitting under flat-band gate voltage conditions [4,13] (Figure 3c). The device with the graphene/Au contacts was estimated to have a significantly lower (0.37 eV) SBH than the Au-only–contacted device (0.47 eV). This decrease in SBH is also accompanied with a reduction in contact resistance (*R*_c_) with respect to that of the Au-only–contacted devices (Figure 3d).

These reduced SBH and *R*_c_ values correlate well with improvement of field-effect mobilities and on/off current ratios estimated from output and transfer characterizations. Wang et al. showed that the insertion of a hexagonal boron nitride film between the 2D channels and metal contacts significantly improves the device performance because of the reduced SBH induced by the interlayer [24,25]. Combining low-work function metals, such as scandium, with our graphene interlayer is expected to further reduce the SBH due to the reduced work function of the graphene interlayer, which will further enhance the device performance. Furthermore, using the dry-transfer method instead of wet-transfer for graphene films might also improve the device’s performance, because the adverse effects of the transfer procedure will be minimized.

**Figure 4:**
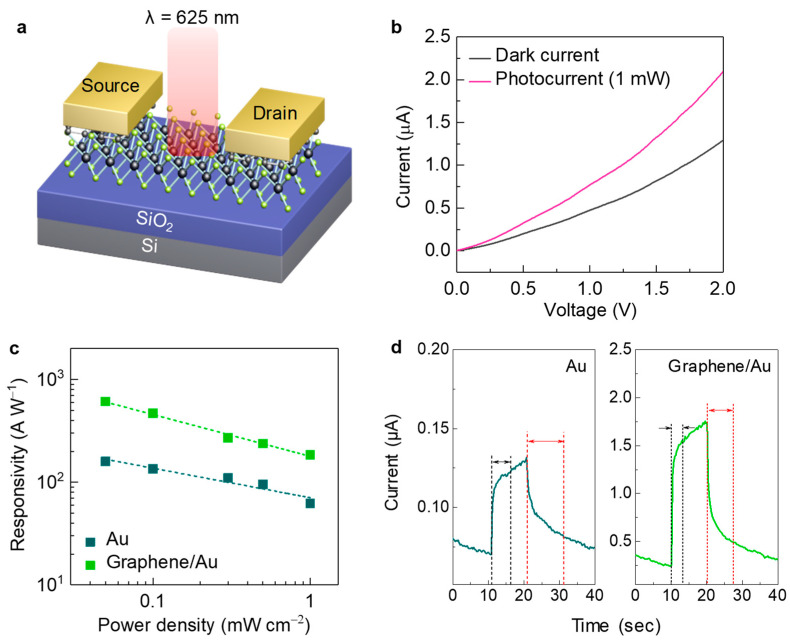
(**a**) Schematic illustration of MoS_2_ device with graphene/Au contacts under visible light illumination (λ = 640 nm). (**b**) *I*-*V* characterizations of MoS_2_ devices in dark or with illumination at 640 nm (*P* = 1 mW). (**c**) Responsivities of MoS_2_ devices with Au and graphene/Au contacts under illumination at 640 nm as a function of light power density. (**d**) Time-dependent photoresponse of MoS_2_ devices.

We finally assessed the photoelectrical properties of MoS_2_ devices under illumination at 640 nm (Figure 4a,b). Under illuminations, the photocurrent occurs in our devices, meaning that photogenerated carriers are created under light illumination conditions. To evaluate the photoelectric performance of our devices, we calculated the light-power dependent responsivity (*R*), defined as *R* = *I*_ph_/(*AP*), where *I*_ph_ is the difference between dark and photocurrents, *A* is the device area, and *P* is the light power density (Figure 4c). Both devices show that the responsivities linearly decrease as the light power density increases. This behavior is attributed to increasing scattering between photogenerated carriers coming from the increased power density [13,26]. This event has been conventionally reported in transition metal dichalcogenide (TMDC)-based photosensor devices [13,26]. Note that the maximal responsivity of the devices with graphene/Au contacts is approximately 610 A/W(±18.3), which is four-fold that of the Au-contacted devices (Figure S2). Furthermore, the achieved responsivity is comparable to that of TMDC photodetector devices reported previously [27,28]. This superior photoelectrical performance of graphene/Au-contacted devices is due to the improved mobility. We also characterized the time-dependent visible photoresponse (λ = 640 nm) of MoS_2_ devices with Au and graphene/Au contacts. Using these graphs, we extracted the rise and decay times, estimated by the change from 10% of the maximum current to 90% and from 90% to 10%, respectively. The graphene/Au-contacted device shows a faster photoresponse (rise time, 3.2 s; decay time, 7.0 s) than those of Au-contacted devices (rise time, 5.2 s; decay time, 12 s). This faster photosensor performance of the graphene/Au-contacted device is due to the efficient carrier separation behavior and low recombination rate caused by the improved carrier mobility [29].

## 4. Conclusions

This report demonstrates the construction of large-area MoS_2_ device arrays with graphene/Au contacts. These graphene/Au-contacted devices have noticeable electrical performance, including a field-effect mobility of 12.06 cm^2^/V∙s and on/off current ratio of 1.0 × 10^7^. This improvement can be attributable to the Fermi-level depinning effect caused by the graphene interlayer between Au and MoS_2_ channels, whereby the SBH and contact resistance are reduced. Furthermore, they show a visible photoresponse, achieving the responsivity of 610 A/W under illumination at 640 nm. This value is significantly comparable to that of TMDC photodetectors. Fabricating large-area MoS_2_ device arrays using graphene films constructed via CVD rather than exfoliated flakes offers insight into practical large-scale optoelectronic applications based on 2D TMDC materials.

## Data Availability

Not applicable.

## Supplementary Materials

The following supporting information can be downloaded at: https://www.mdpi.com/article/10.3390/s22249687/s1, Figure S1: Digital photographs and Raman mapping images of MoS_2_ and graphene films; Figure S2: Average electrical and photoelectrical properties of MoS_2_ devices depending on the type of metal contacts.
