# Supplementary material for: Improvement of Visible Photodetection of Chemical Vapor Deposition-Grown MoS2 Devices via Graphene/Au Contacts
Source: Sensors (Basel). 2022 Dec 10;22(24):9687. doi: 10.3390/s22249687 (PMC9783588; doi:10.3390/s22249687)
Supplement: Supplementary file 1 [file sensors-22-09687-s001.zip › sensors-2084327-supplementary.pdf]

Supplementary Materials

Improvement of Visible Photodetection of Chemical Vapor Deposition-grown MoS_2 Devices via Graphene/Au Contacts

Yeongsik Hwa [1,2] and Sang-Soo Chee [1,*]

[1] Nano Convergence Materials Center, Korea Institute of Ceramic Engineering and Technology (KICET), Jinju, 52851, Republic of Korea

[2] School of Materials Science and Engineering, Gyeongsang National University, Jinju, 52828, Republic of Korea

*Correspondence: sschee@kicet.re.kr (S.S.C.)

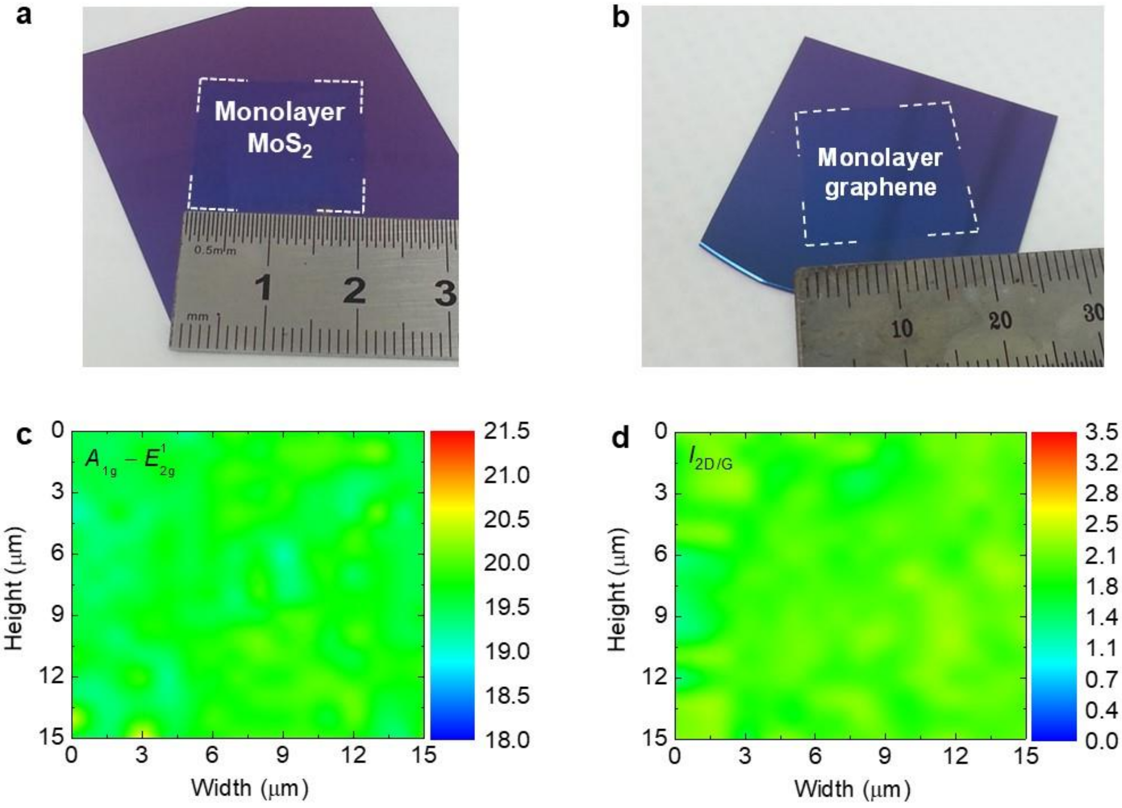

Figure S1. Digital photographs of (a) MoS_2 and (b) graphene films grown by CVD. Raman mapping images of (c) $A_{1g} – E_{2g}$ peak difference and (d) $I_{2D/G}$ for MoS_2 and graphene, respectively.

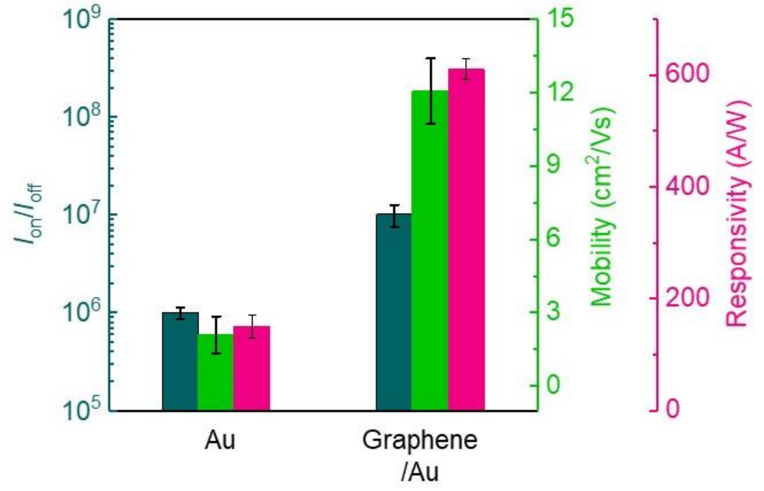

Figure S2. Average mobilities, on/off current ratios, and responsivities of MoS_2 devices with Au and graphene/Au contacts.
